# State and National Estimates of the Cost of Emergency Department Pediatric Readiness and Lives Saved

**DOI:** 10.1001/jamanetworkopen.2024.42154

**Published:** 2024-11-01

**Authors:** Craig D. Newgard, Amber Lin, Jeremy D. Goldhaber-Fiebert, Katherine E. Remick, Marianne Gausche-Hill, Randall S. Burd, Susan Malveau, Jennifer N. B. Cook, Peter C. Jenkins, Stefanie G. Ames, N. Clay Mann, Nina E. Glass, Hilary A. Hewes, Mary Fallat, Apoorva Salvi, Brendan G. Carr, K. John McConnell, Caroline Q. Stephens, Rachel Ford, Marc A. Auerbach, Sean Babcock, Nathan Kuppermann

**Affiliations:** 1 Center for Policy and Research in Emergency Medicine, Department of Emergency Medicine, Oregon Health & Science University, Portland; 2 Department of Health Policy, School of Medicine, Center for Health Policy, Freeman Spogli Institute, Stanford University, Stanford, California; 3 Departments of Pediatrics and Surgery, Dell Medical School, University of Texas at Austin, Austin; 4 Los Angeles County Emergency Medical Services, Harbor-UCLA Medical Center, Torrance, California; 5 Division of Trauma and Burn Surgery, Center for Surgery Care, Children’s National Hospital, Washington, DC; 6 Department of Surgery, Indiana University School of Medicine, Indianapolis; 7 Department of Pediatrics, University of Utah School of Medicine, Salt Lake City; 8 Department of Surgery, Rutgers New Jersey Medical School, Newark; 9 Department of Surgery, University of Louisville School of Medicine, Norton Children’s Hospital, Louisville, Kentucky; 10 Icahn School of Medicine at Mount Sinai, New York, New York; 11 Center for Health Systems Effectiveness, Department of Emergency Medicine, Oregon Health and Science University, Portland; 12 Department of Surgery, University of California, San Francisco; 13 Oregon Emergency Medical Services for Children Program, Oregon Health Authority, Portland; 14 Departments of Pediatrics and Emergency Medicine, Yale University School of Medicine, New Haven, Connecticut; 15 Departments of Emergency Medicine and Pediatrics, University of California, Davis School of Medicine, Sacramento

## Abstract

**Importance:**

High emergency department (ED) pediatric readiness is associated with improved survival among children receiving emergency care, but state and national costs to reach high ED readiness and the resulting number of lives that may be saved are unknown.

**Objective:**

To estimate the state and national annual costs of raising all EDs to high pediatric readiness and the resulting number of pediatric lives that may be saved each year.

**Design, Setting, and Participants:**

This cohort study used data from EDs in 50 US states and the District of Columbia from 2012 through 2022. Eligible children were ages 0 to 17 years receiving emergency services in US EDs and requiring admission, transfer to another hospital for admission, or dying in the ED (collectively termed at-risk children). Data were analyzed from October 2023 to May 2024.

**Exposure:**

EDs considered to have high readiness, with a weighted pediatric readiness score of 88 or above (range 0 to 100, with higher numbers representing higher readiness).

**Main Outcomes and Measures:**

Annual hospital expenditures to reach high ED readiness from current levels and the resulting number of pediatric lives that may be saved through universal high ED readiness.

**Results:**

A total 842 of 4840 EDs (17.4%; range, 2.9% to 100% by state) had high pediatric readiness. The annual US cost for all EDs to reach high pediatric readiness from current levels was $207 335 302 (95% CI, $188 401 692-$226 268 912), ranging from $0 to $11.84 per child by state. Of the 7619 child deaths occurring annually after presentation, 2143 (28.1%; 95% CI, 678-3608) were preventable through universal high ED pediatric readiness, with population-adjusted state estimates ranging from 0 to 69 pediatric lives per year.

**Conclusions and Relevance:**

In this cohort study, raising all EDs to high pediatric readiness was estimated to prevent more than one-quarter of deaths among children receiving emergency services, with modest financial investment. State and national policies that raise ED pediatric readiness may save thousands of children’s lives each year.

## Key Points

**Question:**

What are the state and national costs of raising all emergency departments (EDs) to high pediatric readiness and the potential number of lives saved?

**Findings:**

In this cohort study of 4840 EDs across the US, 842 (17.4%) had high pediatric readiness and the annual cost to reach high pediatric readiness was $207 335 302, ranging from $0 to $11.84 per child by state. An estimated 2143 pediatric lives may be saved each year through universal high ED pediatric readiness.

**Meaning:**

These results suggest that raising all EDs to high pediatric readiness would potentially save thousands of pediatric lives each year, with modest financial investment.

## Teaser

This cohort study of US emergency departments examines the cost effectiveness of high readiness for pediatric cases, estimating costs and lives saved by maintaining universal high pediatric readiness.

## Introduction

Emergency department (ED) pediatric readiness represents the ability to care for acutely ill and injured children, which encompasses care coordination, personnel, quality improvement, safety, policies and procedures, and equipment.^1^ A high level of ED pediatric readiness is associated with improved survival compared with low-readiness EDs among critically ill children,^2^ injured children admitted to trauma centers,^3,4,5^ and children with injuries and medical conditions requiring hospitalization.^6^ The survival benefit persists to 1 year after the ED visit.^3,6^ There is a modestly higher cost of delivering care in high readiness EDs^7^ and the hospital costs needed to reach and sustain high ED readiness.^8^ Raising all EDs in the US to high pediatric readiness is more cost-effective than several routine childhood vaccinations and the level of cost-effectiveness for high ED readiness meets all commonly accepted thresholds.^9,10,11,12^

While evidence for the benefits of high ED pediatric readiness is compelling, a 2023 national assessment^13^ showed that pediatric readiness stagnated from 2013 to 2021 with decreases among EDs that took both assessments. There has also been a steady decline in pediatric inpatient services and consolidation of pediatric admissions in urban tertiary care hospitals over the past decade, resulting in longer travel distances for these services.^14,15^ Combined with known barriers in access to pediatric trauma care,^16,17^ ensuring universal access to high-quality pediatric emergency care is one mechanism to address the limited distribution of pediatric resources and increasing mortality among children in the US.^18^ In this study, we estimated the state-by-state and national costs of raising all EDs to high pediatric readiness from current levels and the resulting annual number of pediatric lives that may be saved.

## Methods

### Study Design

This observational study was reviewed and approved by institutional review boards (IRBs) at Oregon Health and Science University and the University of Utah School of Medicine. The IRBs waived the requirement for informed consent because this study was based on existing data sources and met criteria for a Health Insurance Portability and Accountability Act waiver. We followed the Strengthening the Reporting of Observational Studies in Epidemiology (STROBE) cohort study reporting guidelines. We used several data sources to estimate current levels of ED pediatric readiness across the US, the costs to raise all EDs to high pediatric readiness, the annual number of deaths among children receiving emergency services, and the number of potential lives saved through universal high ED pediatric readiness (eTable 1 and eAppendix 1 in Supplement 1).

### Study Setting

We identified all EDs caring for children in the US, their levels of pediatric readiness and annual volumes of children. Among the 5150 EDs invited to complete the 2021 national pediatric readiness assessment, 3557 had sufficient data to calculate the level of readiness and pediatric volume.^13^ For an additional 1064 EDs that did not complete the 2021 assessment, we used their responses from the 2013 national assessment. We matched EDs to the 2021 American Hospital Association (AHA) annual survey to provide additional information on each facility. We excluded hospitals in US territories, specialty hospitals, hospitals with no ED, EDs without coverage for 24 hours per day and 7 days per week, long-term acute care hospitals, and hospitals with insufficient information to confirm the presence of an ED (eFigure 1 in Supplement 1). For 289 EDs with matched AHA data that did not complete either national readiness assessment, we imputed ED pediatric readiness and annual pediatric volumes using hospital-level multiple imputation^19^ with fully conditional specification.^20,21^ In total, we included 4840 EDs from 50 states and the District of Columbia (eFigure 1 in Supplement 1).

### Patient Population

We included children aged 0 to 17 years receiving emergency services and requiring hospitalization, transferred for hospitalization to a different hospital, or who died in the ED (collectively termed *at-risk children*). Children were followed through the duration of their hospital stay. At-risk children are sensitive to different levels of ED pediatric readiness^2,3,4,5,6^ and represent approximately 3.7% of all children presenting to EDs.^7^

### Emergency Departments and Pediatric Readiness

To measure the level of ED pediatric readiness, we used the weighted Pediatric Readiness Score (wPRS) from 2 recent national assessments that included US EDs providing emergency care 24 hours per day, 7 days per week.^13,22^ The 2021 assessment was completed from May through August 2021 (71% response rate, 3557 of 5150 invited hospitals)^13^ and the 2013 assessment was completed from January through August 2013 (83% response rate, 4146 of 5017 invited hospitals).^22^ The wPRS is a weighted score from 0 to 100 based on questions with moderate-to-high clinical importance in 6 domains,^23^ with 100 representing the highest level of ED pediatric readiness^13,22^ and alignment with national ED guidelines for the care of children.^24^ Calculation of the wPRS was similar in both national assessments. The wPRS has been used to quantify ED pediatric readiness across US hospitals^13,22^ and trauma centers.^4,25^ We matched the national assessments to each ED using hospital name, address, and zip code. The threshold of wPRS associated with improved survival has ranged from 88 to 95 in prior studies.^3,4,5,6^ Because the largest evaluation of ED pediatric readiness (796 937 children cared for in 983 EDs in 11 states) showed that wPRS 88 or higher was associated with increased survival,^6^ we used this level to define high ED pediatric readiness.

### Outcomes

The primary outcomes were state-specific annual costs (adjusted to 2023 US dollars^26^) to reach and sustain high ED pediatric readiness from current levels and the resulting number of pediatric lives that may be saved through high ED pediatric readiness.

### Statistical Analysis

#### Cost Estimations

To estimate the annual state-specific costs to reach and sustain high ED pediatric readiness, we applied previously derived hospital-based annual cost estimates specific to annual ED pediatric volume and current levels of ED readiness^8^ for every ED in each state. To estimate the costs per child in each state, we divided the annual cost to reach high ED readiness by the number of children (ages 0 to 17 years) residing in the state using the 2022 American Community Survey population averages for 2018 to 2022.^27^ We estimated uncertainty in the estimates based on previously calculated means and 95% CIs for hospital-level costs,^8^ with bootstrapped standard errors weighted by the number of EDs in each readiness quartile and volume category (defined below).

#### Estimating Number of Lives Saved

To estimate the annual number of lives that may be saved in each state under a scenario of universal high ED pediatric readiness, we first calculated the annual number of at-risk children in each state. Because standard data sources do not capture these children in every state, we developed a method to estimate state-specific numbers. Based on the cohort of children receiving emergency services in 11 states,^6^ we created a hospital-level distribution of at-risk children by wPRS quartile (0 to 58, 59 to 72, 73 to 87, and 88 to 100) and annual ED volume of children (less than 1800 children; 1800 to 4999 children; 5000 to 9999 children; 10 000 children or more). For each of the 16 combinations of ED pediatric readiness and annual pediatric volume, we calculated the median annual number of at-risk children per ED (eTable 2 in Supplement 1) and applied this distribution to all 4840 US EDs based on known values for pediatric readiness and annual volume. We validated the estimates using 2019 data for 14 states participating in the Health Care Utilization Project and reporting the number of children hospitalized through the ED,^28^ combined with the number of children who died in the ED as available in the Centers for Disease Control and Prevention WONDER database^29^ (eFigure 2 in Supplement 1). We selected 2019 as the most recent year approximating current annual numbers of at-risk children because the COVID-19 pandemic in 2020 temporarily reduced the number of ED visits across the US.^30,31^

Based on the number of at-risk children in each state, we estimated the annual number of deaths among children presenting to EDs under current levels of pediatric readiness by applying the observed probability of death in each of the 16 combinations of pediatric readiness and annual ED pediatric volumes. We estimated the probability of death using the cohort of 796 937 children receiving emergency services in 983 EDs,^6^ with validation of the state-specific death estimates against the 2019 numbers in all 50 states using the WONDER database^21^ (eFigure 3 in Supplement 1). Because WONDER does not separate inpatient deaths by admission source, we used a ratio from 11 states^6^ to estimate the number of inpatient deaths among children admitted through the ED.

To estimate the annual reduction in pediatric deaths (lives saved) in each state under a scenario of universal high ED pediatric readiness, we applied the adjusted reduction in probability of death associated with high ED readiness to the annual number of deaths, accounting for current levels of ED readiness and ED pediatric volumes. To estimate the risk-adjusted reduction in probability of death associated with high ED readiness, we used a hierarchical, mixed effects logistic regression model with a random slope and random intercept to account for clustering by the initial ED separately for children presenting with injuries vs acute medical illness.^6^ We calculated the adjusted predictive margins for quartiles 1 to 3 of ED pediatric readiness vs the counterfactual scenario of high ED readiness (quartile 4), plus the corresponding standard errors. The difference between the annual number of deaths at current levels of ED pediatric readiness vs universal high ED readiness yielded the estimated number of lives that may be saved by state. We summed the state-specific estimates to generate a national estimate and calculated uncertainty using the standard errors of observed and counterfactual probabilities of death with bootstrapping to generate 95% CIs.^32^ Details of the methods and data sources used to generate state and national estimates for costs and lives saved are included in Supplement 1. We used data from 2012 through 2022, which we analyzed between October 2023 to May 2024.

We used SAS version 9.4 (SAS Institute Inc) for data management, analysis, and imputation, Stata version 18 (StataCorp) for model-based estimates of mortality reduction, and R version 4.3.2 (R Foundation for Statistical Computing) for graphing. All statistical tests were 2-sided with an α < .05.

## Results

**Figure 1.**
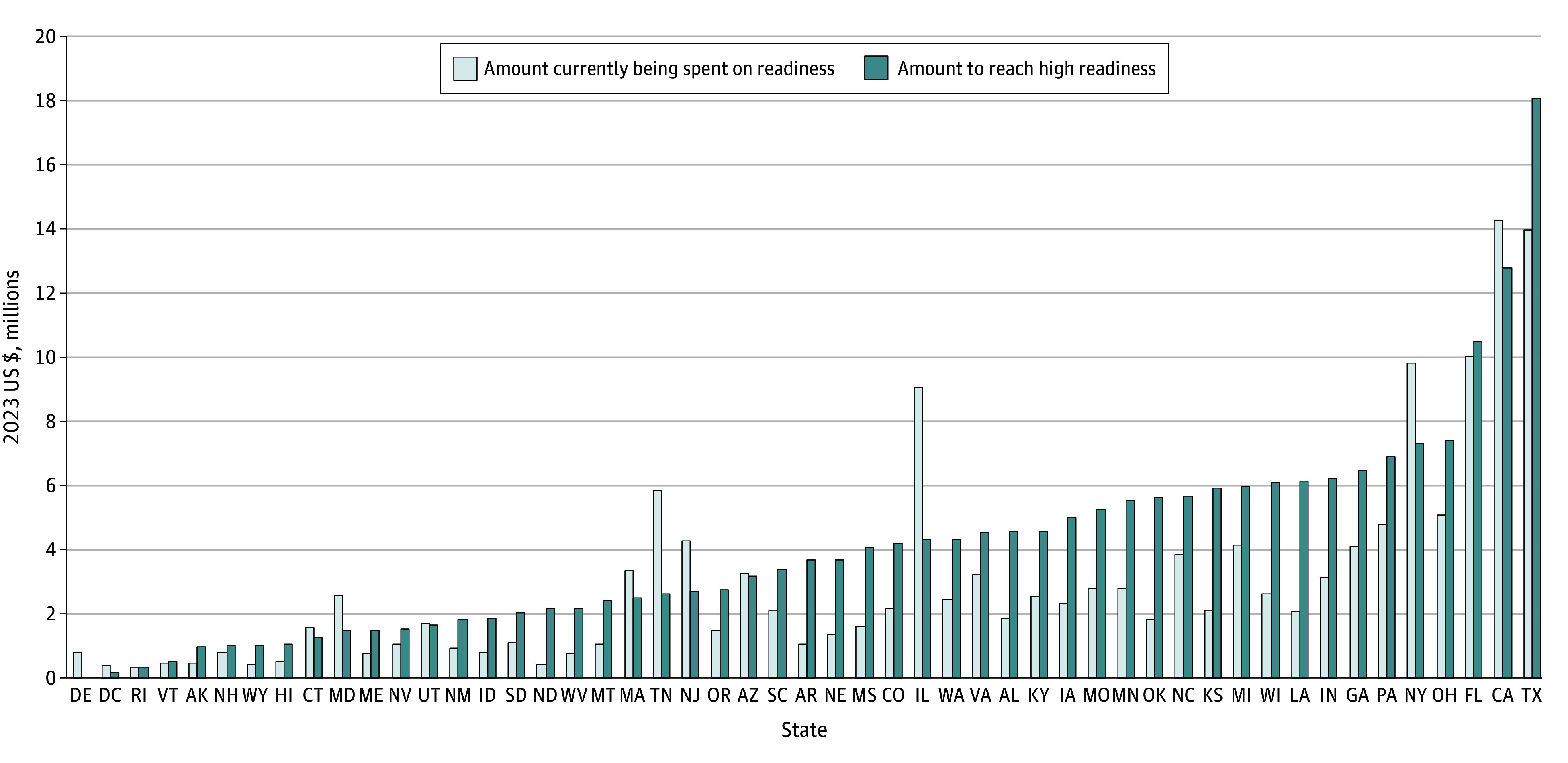
Current Annual Investment in ED Pediatric Readiness and the Annual Cost to Reach High Readiness by State

**Figure 2.**
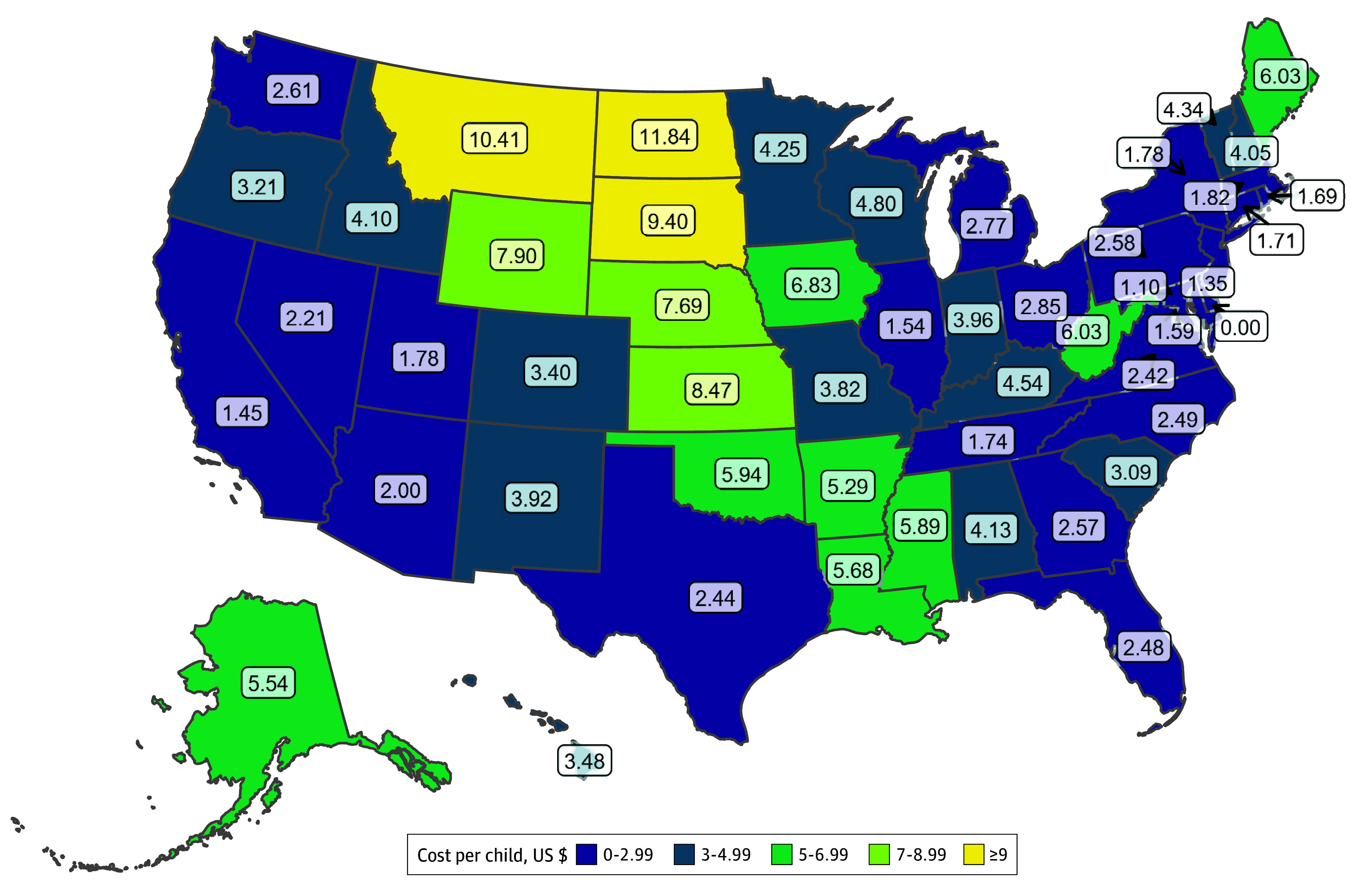
Annual Cost per Child Resident to Reach Universal High ED Pediatric Readiness by State Costs per child were calculated based on the total pediatric (ages 0-17 years) population in each state.

We estimated that 669 019 at-risk children receive emergency services in 4840 EDs across the US each year (Table). Among the 4840 EDs, the median (IQR) wPRS was 69 (59-82; range, 26-100). Based on current levels of ED pediatric readiness, 842 of 4840 EDs (17.4%) had high readiness (ie, wPRS 88 or above) with a range of 2.9% in Arkansas to 100% in Delaware. The annual cost for all EDs in the US to reach high ED pediatric readiness from current levels was $207 335 302 (95% CI, $188 401 692-$226 268 912), ranging from $0 in Delaware to $18 045 405 in Texas (Table). Current annual investment in ED pediatric readiness and the cost to reach high ED readiness are shown in Figure 1. The annual costs per child to reach universal high ED pediatric readiness ranged from $0 in Delaware to $11.84 in North Dakota (Figure 2).

**Table.** State and National Estimates of Emergency Departments, Children Served, Deaths in the Context of Pediatric Readiness, and Costs to Reach High Pediatric Readiness

States	No. of EDs caring for children, No. (%)	Annual at-risk ED visits by children, No. (%)^a^	Annual child deaths given current level of ED readiness, No. (%)	Annual child deaths if all EDs had high pediatric readiness, No. (%)	Annual child lives that could be saved if all EDs had high pediatric readiness, No. (%)	Annual cost to reach high ED readiness, $ (%)
US total	4840	669 019	7619	5476	2143	207 335 302
Alabama	89 (1.8)	7611 (1.1)	110 (1.4)	63 (1.2)	47 (2.2)	4 567 670 (2.2)
Alaska	23 (0.5)	993 (0.1)	15 (0.2)	7 (0.1)	8 (0.4)	993 108 (0.5)
Arizona	79 (1.6)	14 730 (2.2)	159 (2.1)	120 (2.2)	39 (1.8)	3 191 078 (1.5)
Arkansas	68 (1.4)	4317 (0.6)	68 (0.9)	33 (0.6)	35 (1.6)	3 687 004 (1.8)
California	332 (6.9)	62 122 (9.3)	659 (8.6)	506 (9.2)	153 (7.1)	12 764 775 (6.2)
Colorado	88 (1.8)	11 313 (1.7)	132 (1.7)	90 (1.6)	42 (2.0)	4 216 015 (2.0)
Connecticut	36 (0.7)	8332 (1.2)	87 (1.1)	71 (1.3)	16 (0.7)	1 267 156 (0.6)
Delaware	10 (0.2)	2369 (0.4)	25 (0.3)	25 (0.5)	0	0
District of Columbia	7 (0.1)	3071 (0.5)	30 (0.4)	28 (0.5)	2 (0.1)	197 767 (0.1)
Florida	253 (5.2)	50 347 (7.5)	547 (7.2)	418 (7.6)	129 (6.0)	10 501 726 (5.1)
Georgia	133 (2.7)	25 665 (3.8)	282 (3.7)	210 (3.8)	72 (3.4)	6 461 980 (3.1)
Hawaii	24 (0.5)	1090 (0.2)	20 (0.3)	9 (0.2)	11 (0.5)	1 061 408 (0.5)
Idaho	40 (0.8)	1499 (0.2)	31 (0.4)	13 (0.2)	18 (0.8)	1 866 838 (0.9)
Illinois	184 (3.8)	25 118 (3.8)	274 (3.6)	239 (4.4)	35 (1.6)	4 334 009 (2.1)
Indiana	134 (2.8)	10 667 (1.6)	143 (1.9)	89 (1.6)	54 (2.5)	6 243 104 (3.0)
Iowa	118 (2.4)	5883 (0.9)	88 (1.2)	57 (1.0)	31 (1.4)	4 989 895 (2.4)
Kansas	131 (2.7)	5703 (0.9)	86 (1.1)	47 (0.9)	39 (1.8)	5 938 061 (2.9)
Kentucky	99 (2.0)	11 123 (1.7)	139 (1.8)	97 (1.8)	42 (2.0)	4 591 956 (2.2)
Louisiana	109 (2.3)	12 459 (1.9)	151 (2.0)	86 (1.6)	65 (3.0)	6 151 792 (3.0)
Maine	35 (0.7)	1327 (0.2)	25 (0.3)	10 (0.2)	15 (0.7)	1 512 136 (0.7)
Maryland	49 (1.0)	11 608 (1.7)	116 (1.5)	93 (1.7)	23 (1.1)	1 496 515 (0.7)
Massachusetts	64 (1.3)	19 487 (2.9)	202 (2.7)	164 (3.0)	38 (1.8)	2 494 113 (1.2)
Michigan	137 (2.8)	18 786 (2.8)	219 (2.9)	155 (2.8)	64 (3.0)	5 958 542 (2.9)
Minnesota	130 (2.7)	8346 (1.2)	107 (1.4)	67 (1.2)	40 (1.9)	5 550 742 (2.7)
Mississippi	83 (1.7)	5691 (0.9)	79 (1.0)	42 (0.8)	37 (1.7)	4 071 543 (2.0)
Missouri	111 (2.3)	13 749 (2.1)	159 (2.1)	111 (2.0)	48 (2.2)	5 254 258 (2.5)
Montana	59 (1.2)	1377 (0.2)	28 (0.4)	14 (0.3)	14 (0.7)	2 407 609 (1.2)
Nebraska	84 (1.7)	3065 (0.5)	50 (0.7)	28 (0.5)	22 (1.0)	3 694 226 (1.8)
Nevada	34 (0.7)	6113 (0.9)	65 (0.9)	42 (0.8)	23 (1.1)	1 525 894 (0.7)
New Hampshire	28 (0.6)	2509 (0.4)	30 (0.4)	22 (0.4)	8 (0.4)	1 037 750 (0.5)
New Jersey	70 (1.4)	27 228 (4.1)	256 (3.4)	205 (3.7)	51 (2.4)	2 711 031 (1.3)
New Mexico	39 (0.8)	3551 (0.5)	46 (0.6)	29 (0.5)	17 (0.8)	1 855 436 (0.9)
New York	186 (3.8)	65 113 (9.7)	627 (8.2)	507 (9.3)	120 (5.6)	7 338 606 (3.5)
North Carolina	120 (2.5)	20 372 (3.0)	229 (3.0)	166 (3.0)	63 (2.9)	5 690 537 (2.7)
North Dakota	44 (0.9)	811 (0.1)	17 (0.2)	7 (0.1)	10 (0.5)	2 163 254 (1.0)
Ohio	162 (3.3)	25 660 (3.8)	294 (3.9)	210 (3.8)	84 (3.9)	7 391 500 (3.6)
Oklahoma	115 (2.4)	6826 (1.0)	95 (1.2)	50 (0.9)	45 (2.1)	5 642 392 (2.7)
Oregon	59 (1.2)	6208 (0.9)	78 (1.0)	49 (0.9)	29 (1.4)	2 748 889 (1.3)
Pennsylvania	152 (3.1)	19 352 (2.9)	238 (3.1)	165 (3.0)	73 (3.4)	6 885 955 (3.3)
Rhode Island	9 (0.2)	1803 (0.3)	18 (0.2)	15 (0.3)	3 (0.1)	352 041 (0.2)
South Carolina	67 (1.4)	11 572 (1.7)	141 (1.9)	103 (1.9)	38 (1.8)	3 414 640 (1.6)
South Dakota	54 (1.1)	1071 (0.2)	23 (0.3)	8 (0.1)	15 (0.7)	2 035 263 (1.0)
Tennessee	114 (2.4)	19 834 (3.0)	212 (2.8)	185 (3.4)	27 (1.3)	2 655 495 (1.3)
Texas	428 (8.8)	60 963 (9.1)	691 (9.1)	495 (9.0)	196 (9.1)	18 045 405 (8.7)
Utah	49 (1.0)	4603 (0.7)	54 (0.7)	37 (0.7)	17 (0.8)	1 666 549 (0.8)
Vermont	14 (0.3)	1088 (0.2)	13 (0.2)	8 (0.1)	5 (0.2)	506 739 (0.2)
Virginia	94 (1.9)	17 219 (2.6)	190 (2.5)	135 (2.5)	55 (2.6)	4 546 805 (2.2)
Washington	90 (1.9)	8993 (1.3)	112 (1.5)	61 (1.1)	51 (2.4)	4 345 589 (2.1)
West Virginia	46 (1.0)	2520 (0.4)	36 (0.5)	16 (0.3)	20 (0.9)	2 169 785 (1.0)
Wisconsin	131 (2.7)	7389 (1.1)	115 (1.5)	66 (1.2)	49 (2.3)	6 095 491 (2.9)
Wyoming	26 (0.5)	373 (0.1)	8 (0.1)	3 (0.1)	5 (0.2)	1 045 230 (0.5)

^a^ Children were defined as at-risk if they received care in emergency departments (EDs) and required admission, transfer to another hospital for admission, or died in the ED.

**Figure 3.**
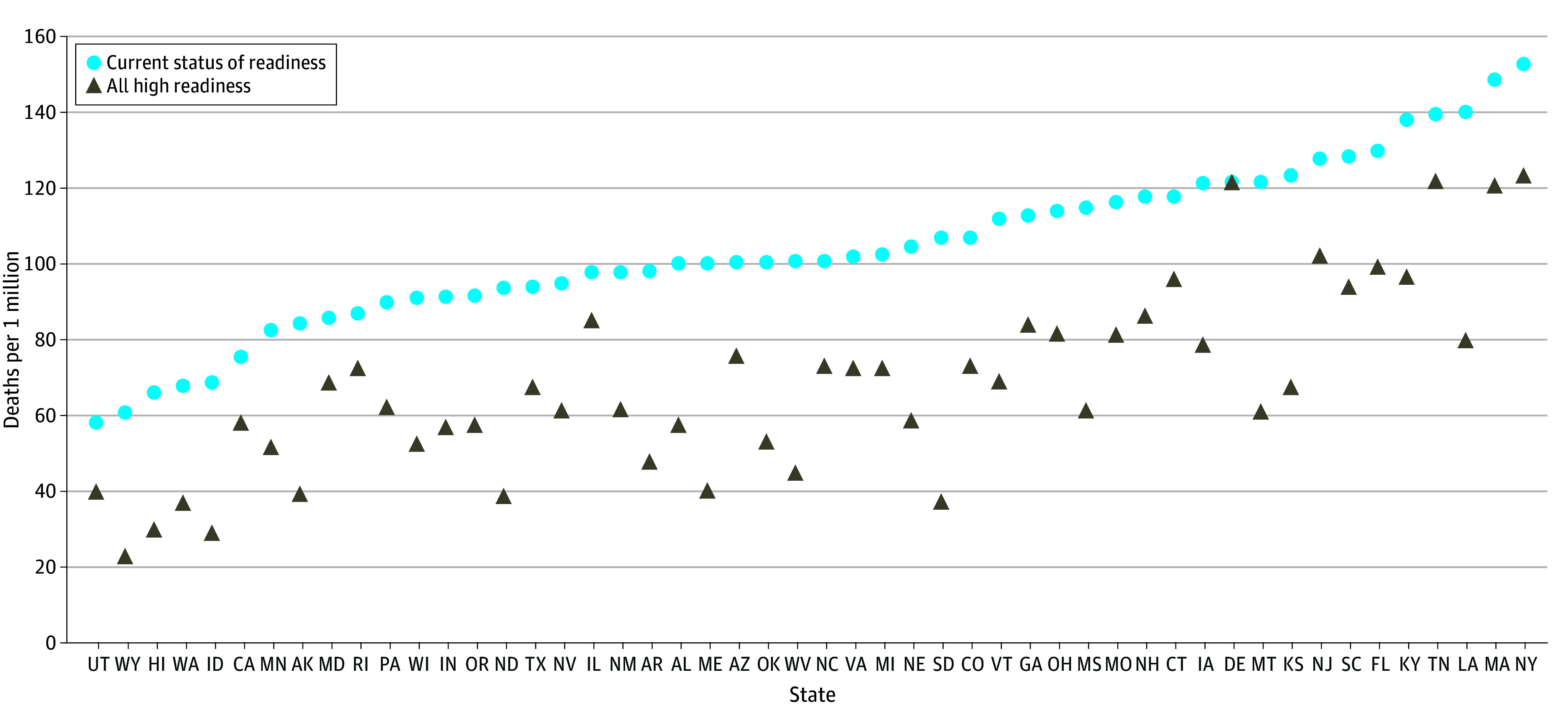
Population-Adjusted Estimates of the Annual Number of Pediatric Deaths Under Current Levels of ED Pediatric Readiness vs Universal High ED Readiness by State Washington, DC is excluded because many children cared for in District hospitals came from outside the District, creating an artificially high population-adjusted estimate based on children residing in the District.

**Figure 4.**
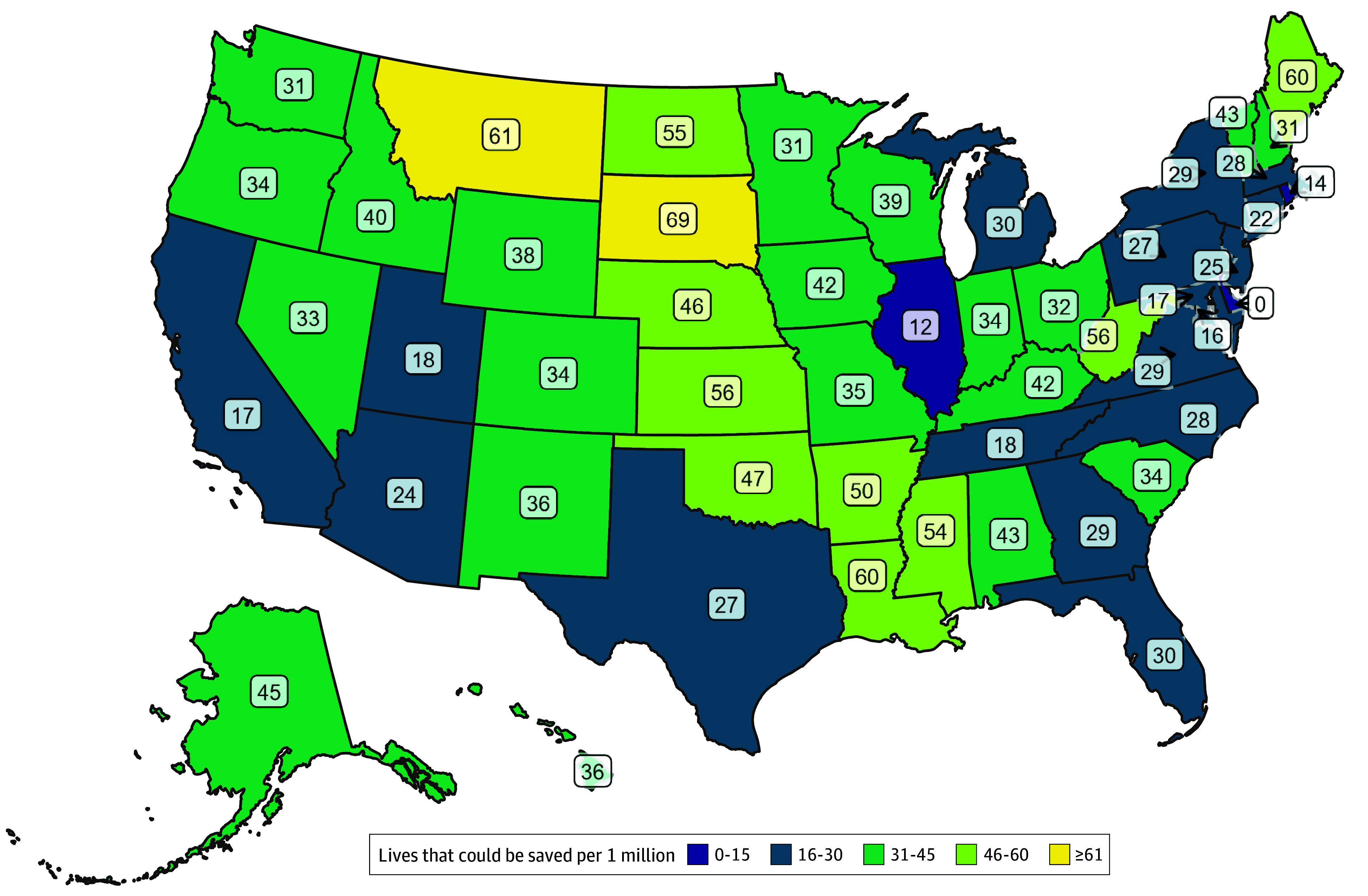
Population-Adjusted Estimates for the Annual Number of Pediatric Lives Saved Through Universal High ED Pediatric Readiness by State

Among the 669 019 at-risk children cared for in EDs each year, we estimated that 7619 (1.1%) die during the acute care visit, including ED and inpatient deaths. The annual number of pediatric deaths under current levels of ED readiness ranged from 8 in Wyoming to 691 in Texas (Table). We estimated that 2143 (28.1%; 95% CI, 678-3608) of the 7619 childhood deaths each year may be preventable through universal high ED pediatric readiness, ranging from zero in Delaware (100% of EDs currently at high readiness) to 507 in New York (25.8% of EDs currently at high readiness) (Table). Figure 3 shows the number of pediatric deaths under current vs universal high ED readiness after accounting for the number of children residing in each state. Population-adjusted estimates for the number of additional pediatric lives that may be saved varied from zero in Delaware to 69 in South Dakota (Figure 4).

The estimates were robust to sensitivity analyses evaluating heterogeneity in the association of high ED pediatric readiness with survival across states. These analyses were also consistent when using 2013 estimates for ED readiness when 2021 values were missing (detailed in eAppendix 2 in Supplement 1).

## Discussion

We estimated the state and national costs to reach universal high ED pediatric readiness and the number of pediatric lives that may be saved through this investment. The state-specific costs per child resident to raise all EDs to high readiness were modest, ranging from $0 to $12 per year. This study adds to the growing body of research demonstrating the association between high ED pediatric readiness and increased survival in children,^2,3,4,5,6^ small incremental costs of delivering care in high-readiness EDs,^7^ modest hospital expenditures to reach high ED readiness,^8^ and the cost effectiveness of raising all EDs in the US to high readiness ($9300 per quality-adjusted life-years gained, $244 000 per life saved).^12^ These results provide policy makers, states, and national organizations the information needed to potentially save thousands of children’s lives each year and to address inequities in access to high quality emergency services for children.

Our results suggest that more than one quarter of deaths among children receiving emergency care in the US may be preventable through ED pediatric readiness. The magnitude of benefit is explained in part by the time to death among children who die after presenting for emergency care. Among children presenting to EDs who died within 1 year, death most commonly occurred at the initial ED^33^ and often within hours of arrival.^3^ That is, the window to intervene is early and narrow. Based on the way that ED pediatric readiness is organized and conceptualized, any hospital can achieve high ED pediatric readiness, regardless of inpatient services or hospital type. The opportunity for all EDs to reach high pediatric readiness allows small EDs in rural regions to provide the same level of high-quality emergency care to children as large tertiary care hospitals in urban settings. When our results are combined with the known decreases in pediatric inpatient services over the past decade^14,15^ and large regions without immediate access to pediatric trauma care,^16,17^ it is clear that there are large health care deserts across the US where children lack access to high-quality acute pediatric care. Increasing the number of high-readiness EDs would partly reduce the number and size of these deserts. The impact of high ED pediatric readiness may be magnified in the setting of disasters, mass casualty incidents, pandemics, and other events that further strain the emergency care system.

Strategies for implementing our findings include a combination of regulation, incentives, and policy-based initiatives. Hospital accreditation organizations could require all hospitals with EDs to have high pediatric readiness. For example, the American College of Surgeons Committee on Trauma added completion of the ED pediatric readiness assessment to the trauma center verification criteria.^34^ Medicaid and private insurance reimbursement for ED pediatric visits could be modified to incentivize hospitals to reach high ED pediatric readiness, using pay-for-performance and value-based purchasing practices^35^ to improve pediatric emergency care. In addition, state pediatric recognition programs that encourage EDs to raise pediatric readiness have been associated with higher levels of readiness.^36^ Federal programs to improve hospital preparedness^37^ and specify conditions of participation (ie, Centers for Medicare and Medicaid Services) could formally integrate ED pediatric readiness. Policies seeking to raise ED pediatric readiness should incentivize adding Pediatric Emergency Care Coordinators (PECCs), who are integral to all aspects of ED readiness^38^ and associated with high observed-to-expected pediatric survival in US trauma centers.^39^ In all but the busiest EDs, PECCs are part-time positions filled by existing nurses and physicians.^8,40^

An equally important question to health policy is who should bear the costs of raising ED pediatric readiness. Saving children’s lives benefits families, communities, and society at large, which suggests the need for societal investment. High ED pediatric readiness is highly cost effective, demonstrating the value of such an investment.^12^ We quantify the costs to reach high ED pediatric readiness at the hospital level, but funding this effort could be operationalized in a number of ways. Plausible funding options include state and federal subsidies, public and private insurers, regionalized health care systems, taxpayer initiatives, and reimbursement incentives. Ideally, funding ED pediatric readiness would be done at the federal level to avoid the uneven patchwork of health care services that already exists across states.

### Limitations

This study has limitations. To generate state-specific estimates for lives saved, we applied an 11-state distribution of at-risk children and pediatric deaths generated from previous research to all states based on known levels of readiness and ED volumes. While it is possible that children receiving emergency care in the 11 states differed from children in other states, external validation of these estimates showed good agreement and suggested the estimates were conservative. As the number of children requiring emergency services returns to and eventually exceeds pre-COVID pandemic levels, the number of lives saved from high ED pediatric readiness will exceed our estimates. Sensitivity analyses showed our results to be robust to these assumptions.

Analysis reflects the state of ED pediatric readiness in 2021. For hospitals that did not complete the 2021 assessment, we used their 2013 information (22% of all EDs in the sample). Given the stagnation of ED readiness from 2013 to 2021,^13^ the sensitivity analyses showed that this strategy was robust and valid, but it is possible that ED readiness could have changed in some hospitals. We used quartiles of wPRS and categories of ED pediatric volume based on previous outcome studies^2,3,4,5,6^ and the national assessments,^13,22^ which decreased granularity in the association between ED readiness and mortality, but allowed for sufficient sample sizes across all states. In addition, we estimated the hospital costs to reach high ED readiness based on previous research^8^ and the number of inpatient deaths among children admitted through the ED based on a ratio from our previous study.^6^ Finally, there may be inherent differences between EDs with high vs low pediatric readiness and between hospitals in each state (ie, unmeasured confounding) that could have affected our mortality reduction estimates. However, our estimates were consistent with similar research done in different states^2^ and across different hospitals.^3,4,5^

## Conclusions

In summary, we quantified the state-specific costs to reach universal high ED pediatric readiness and showed that this investment may prevent more than one quarter of deaths among children receiving emergency services in the US each year. The costs and number of lives saved varied across states, but consistently showed large benefits for low costs per child resident. These estimates provide policy makers the necessary information to enhance the US pediatric emergency care system and save lives.

**Accepted for Publication:** August 27, 2024.

## Notes

Supplement 1. **eTable 1.** Data Sources, Years, and Variables Used for the Study **eFigure 1.** Schematic of Emergency Department Cohort Creation **eTable 2.** Median Annual Number of At-Risk Children by Weighted Pediatric Readiness Score (wPRS) Quartile and Emergency Department (ED) Pediatric Volume Across 11 States **eFigure 2.** State-level Comparison of Estimated Versus Actual Annual Numbers of At-Risk Children Receiving Care in Emergency Departments in 14 States in 2019 **eFigure 3.** State-level Comparison of Estimated Versus Actual Annual Number of Deaths Among Children Presenting to EDs in 50 States in 2019 **eAppendix 1.** Details of the Statistical Analysis **eAppendix 2.** Description of Sensitivity Analysis
eReferences

Supplement 2.
Data Sharing Statement
