# Supplementary material for: State and National Estimates of the Cost of Emergency Department Pediatric Readiness and Lives Saved
Source: JAMA Netw Open. 2024 Nov 1;7(11):e2442154. doi: 10.1001/jamanetworkopen.2024.42154 (PMC11530936; doi:10.1001/jamanetworkopen.2024.42154)
Supplement: Supplement 1. — eTable 1. Data Sources, Years, and Variables Used for the Study eFigure 1. Schematic of Emergency Department Cohort Creation eTable 2. Median Annual Number of At-Risk Children by Weighted Pediatric Readiness Score (wPRS) Quartile and Emergency Department (ED) Pediatric Volume Across 11 States eFigure 2. State-level Comparison of Estimated Versus Actual Annual Numbers of At-Risk Children Receiving Care in Emergency Departments in 14 States in 2019 eFigure 3. State-level Comparison of Estimated Versus Actual Annual Number of Deaths Among Children Presenting to EDs in 50 States in 2019 eAppendix 1. Details of the Statistical Analysis eAppendix 2. Description of Sensitivity Analysis eReferences [file jamanetwopen-e2442154-s001.pdf]

Supplemental Online Content

Newgard CD, Lin A, Goldhaber-Fiebert JD, et al. State and National Estimates of the Cost of Emergency Department Pediatric Readiness and Lives Saved. *JAMA Netw Open.* 2024;7(11):e2442154. doi:10.1001/jamanetworkopen.2024.42154

eTable 1. Data Sources, Years, and Variables Used for the Study

eFigure 1. Schematic of Emergency Department Cohort Creation

eTable 2. Median Annual Number of At-Risk Children by Weighted Pediatric Readiness Score (wPRS) Quartile and Emergency Department (ED) Pediatric Volume Across 11 States

eFigure 2. State-level Comparison of Estimated Versus Actual Annual Numbers of At-Risk Children Receiving Care in Emergency Departments in 14 States in 2019

eFigure 3. State-level Comparison of Estimated Versus Actual Annual Number of Deaths Among Children Presenting to EDs in 50 States in 2019

eAppendix 1. Details of the Statistical Analysis

eAppendix 2. Description of Sensitivity Analysis

eReferences.

This supplemental material has been provided by the authors to give readers additional information about their work.

© 2024 Newgard CD et al. *JAMA Network Open*.

eTable 1. Data sources, years, and variables used for the study.

Data Source	Years used	Variables used	Purpose	Description and References
National Pediatric Readiness Project Assessments	2021 & 2013	Weighted pediatric readiness score (wPRS) and annual ED pediatric volume	Primary predictor	Two national assessments of EDs using a structured survey instrument that collected information on ED pediatric readiness across 6 domains and used to generate a global score of ED readiness (the wPRS).<br><br>Remick KE, Hewes HA, Ely M, et al. National Assessment of Pediatric Readiness of US Emergency Departments During the COVID-19 Pandemic. *JAMA Netw Open*. Jul 3 2023;6(7):e2321707.<br><br>Gausche-Hill M, Ely M, Schmuhl P, et al. A national assessment of pediatric readiness of emergency departments. *JAMA Pediatr*. Jun 2015;169(6):527-34. doi:10.1001/jamapediatrics.2015.138
State ED and inpatient data	2012-2017	Patient demographics, clinical characteristics, and mortality.	Estimates of mortality reduction of high ED pediatric readiness	Cohort of children compiled from State Emergency Department Data (SEDD) and State Inpatient Data (SID) sources for 983 EDs in 11 states from 2012-2017, including demographics, acuity and severity, procedures, diagnoses, and outcomes.<br><br>Newgard CD, Lin A, Malveau S, et al. Emergency Department Pediatric Readiness and Short-term and Long-term Mortality Among Children Receiving Emergency Care. *JAMA Netw Open.* 2023;6(1):e2250941
Costs to reach high ED pediatric readiness	2022	Hospital cost of ED pediatric readiness	Outcome	The study quantified the total annual cost of high ED pediatric readiness based on national labor costs, a sample of 983 EDs, the 2021 national ED pediatric readiness assessment, two PECC surveys, and medical supply cost data from 7 healthcare organizations. The cost to reach high ED readiness was estimated based on ED pediatric volume (5 categories) and current levels of ED readiness from the 2021 national assessment.<br><br>Remick KE, Gausche-Hill M, Lin A, Goldhaber-Fiebert JD, Lang B, Foster A, Burns B, Jenkin, PC, Hewes HA, Kupperman, N, McConnell KJ, Marin J, Weyant C, Ford R, Babcock SR, & Newgard CD. The hospital costs of high emergency department pediatric readiness. JACEP Open, 2024 June 03: 5(3), e13179.
American Hospital Association data	2021	ED and hospital characteristics	Cohort creation and multiple imputation of missing wPRS	Data on hospital characteristics, ownership, volume, and services collected for 6,120 hospitals across the U.S. each year.<br><br>https://www.ahadata.com/aha-annual-survey-database
HCUPNet	2019		Validation of number of "at-risk"	The AHRQ Healthcare Cost and Utilization Project provides a series of web tools that can be used to

© 2024 Newgard CD et al. *JAMA Network Open*.

			children in the ED by state	calculate the number of admissions, deaths, and other statistics on participating hospitals across the U.S.<br><br>https://datatools.ahrq.gov/hcupnet/
WONDER	2019		Validation of number of ED deaths by state	The WONDER database is maintained by the CDC and provides an on-line method for calculating the number and location of deaths by age for individual states.<br><br>https://wonder.cdc.gov/controller/datarequest/D158
American Community Survey	2018-2022 (5-year average)	Children (0-17 years) residents per state	Creation of cost-per-child and lives saved rate by state	Census survey of the U.S. population.<br><br>https://www.census.gov/programs-surveys/acs/about.html

© 2024 Newgard CD et al. *JAMA Network Open*.

eFigure 1. Schematic of emergency department cohort creation.

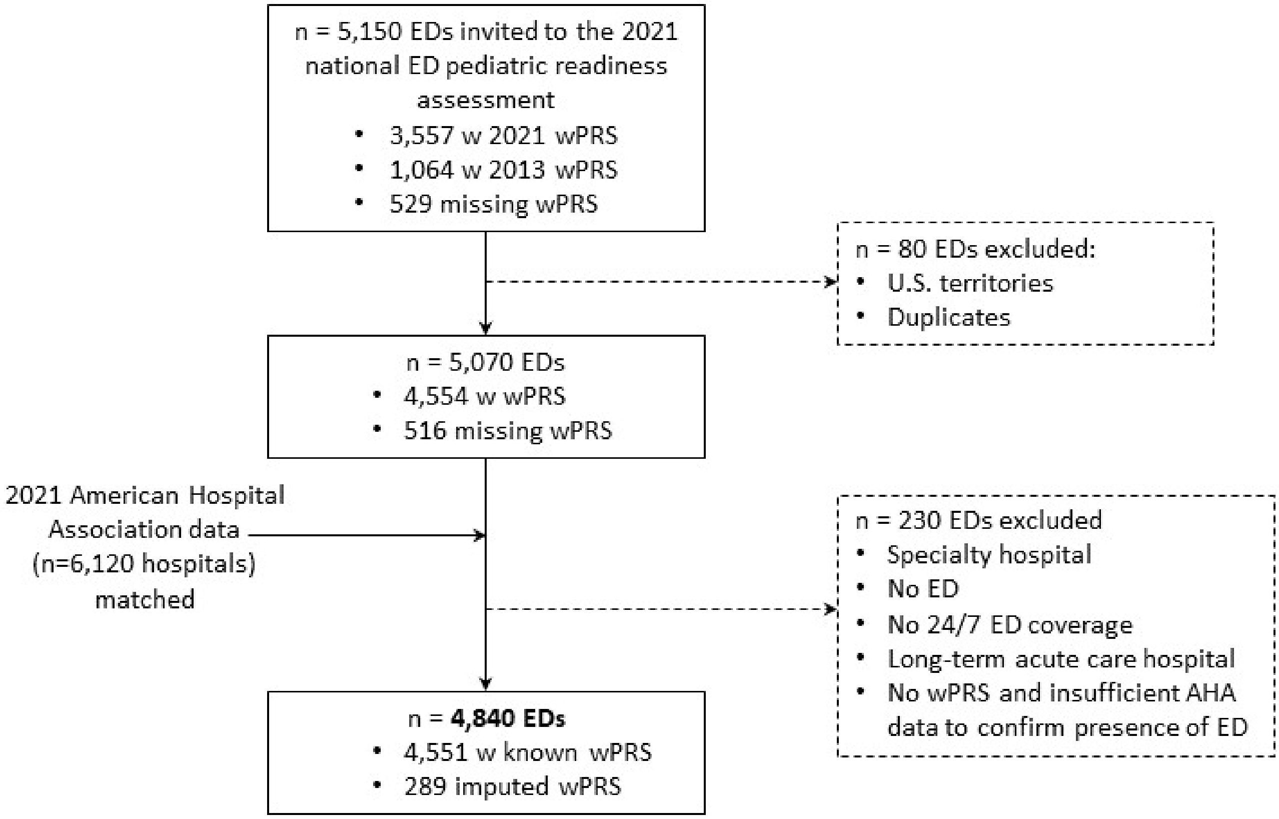

© 2024 Newgard CD et al. *JAMA Network Open*.

eTable 2. Median annual number of at-risk children by weighted pediatric readiness score (wPRS) quartile and emergency department (ED) pediatric volume across 11 states.

wPRS quartile	Annual ED pediatric volume category	n EDs	Median annual ED visits for "at risk" children
1st (lowest)	Low	125	8
2nd	Low	161	8
3rd	Low	87	8
4th (highest)	Low	33	4
1st (lowest)	Medium	50	51
2nd	Medium	136	60
3rd	Medium	87	48
4th (highest)	Medium	74	87
1st (lowest)	Medium High	15	129
2nd	Medium High	44	124
3rd	Medium High	50	131
4th (highest)	Medium High	52	347
1st (lowest)	High	5	226
2nd	High	16	259
3rd	High	36	1018
4th (highest)	High	85	1495

*"At-risk" children were those whose ED visits resulted in hospitalization, transfer to another hospital for hospitalization, or death.

© 2024 Newgard CD et al. *JAMA Network Open*.

eFigure 2. State-level comparison of estimated versus actual annual numbers of at-risk children receiving care in emergency departments in 14 states in 2019.*

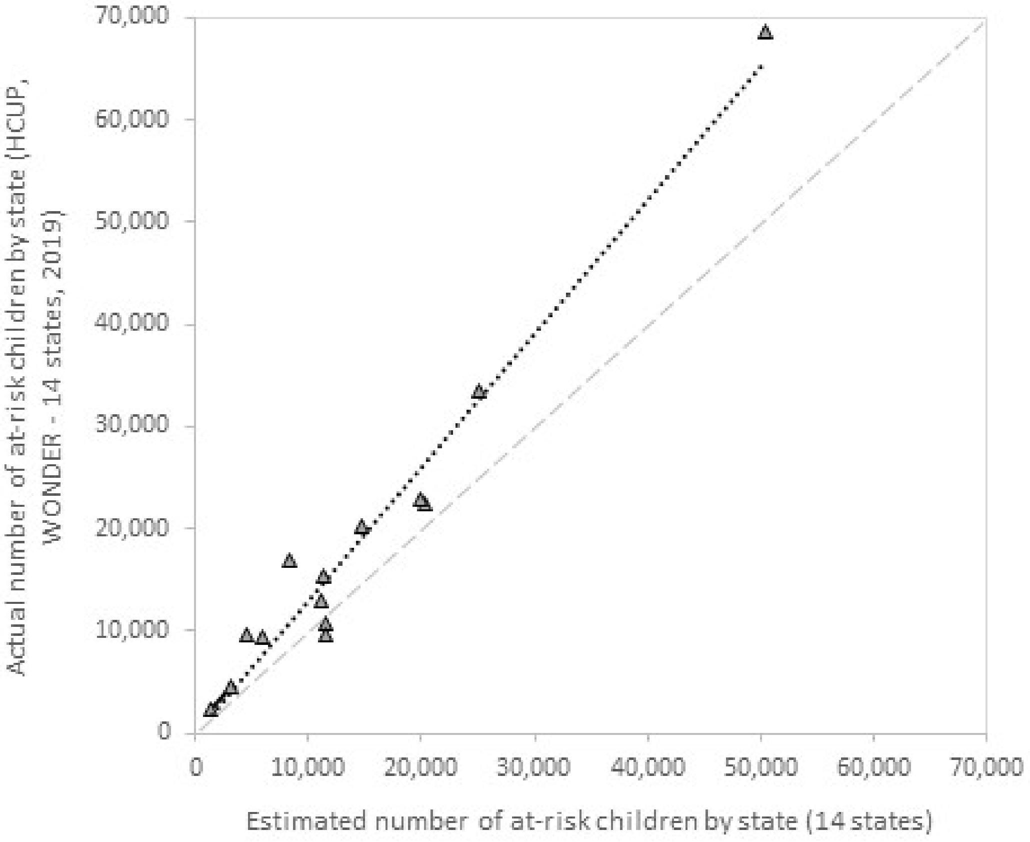

*Grey dashed line represents perfect agreement; black dotted line represents linear line fitted to the data. At-risk children defined as children presenting to EDs who required hospitalization, transfer to another hospital for hospitalization, or died in the ED. The 14 states included: Arizona, Colorado, Florida, Illinois, Iowa, Kentucky, Maryland, Minnesota, Montana, Nebraska, North Carolina, South Carolina, Tennessee, and Utah.

© 2024 Newgard CD et al. *JAMA Network Open*.

eFigure 3. State-level comparison of estimated versus actual annual number of deaths among children presenting to EDs in 50 states in 2019.*

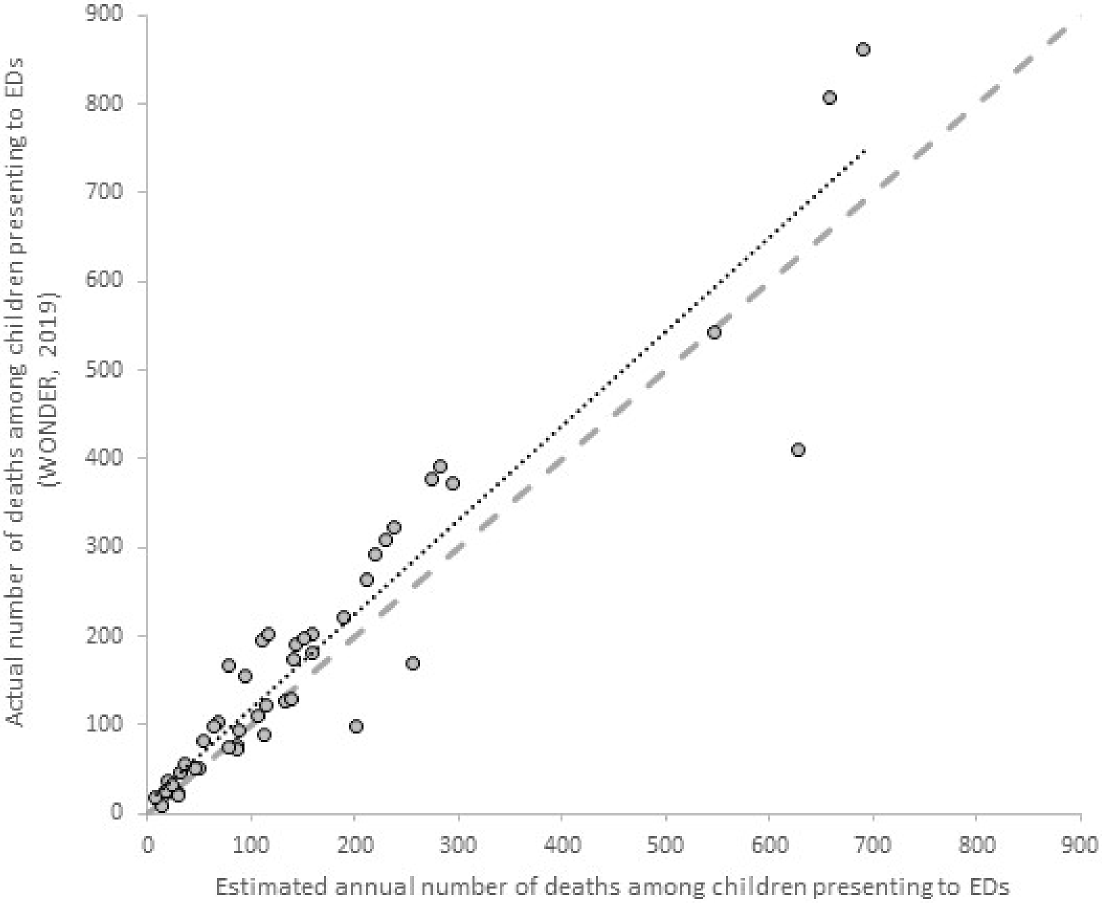

*Grey dashed line represents perfect agreement; black dotted line represents linear line fitted to the data.

© 2024 Newgard CD et al. *JAMA Network Open*.

eAppendix 1. Details of the statistical analysis.

1. **Definitions**
    a. Included states (11 states with patient-level emergency department [ED] and inpatient data matched to the level of ED pediatric readiness[1]): Arizona, California, Florida, Iowa, Maryland, Minnesota, New Jersey, North Carolina, New York, Rhode Island, and Wisconsin.
    b. Emergency Departments (EDs): For this analysis, we are considering verified EDs open 24/7 in 50 states and the District of Columbia.
    c. Weighted Pediatric Readiness Score (wPRS) quartiles: Weighted Pediatric Readiness Score (a global measure of ED pediatric readiness from the national assessment, range 0-100 with higher numbers denoting higher levels of readiness), categorized into quartiles based on prior research[1] - Quartile 1 (0-58, lowest); Quartile 2 (59-72); Quartile 3 (73-87); Quartile 4 (88-100, highest). A wPRS in the highest quartile is associated with improved survival in children.[1]
    d. Injury versus medical ED visits: All estimates and analyses were performed separately for injury and medical cohorts (based on ICD9/10 diagnoses from the index ED visit/admission) due to the inherent differences in modeling mortality and the risk-adjusted association between ED readiness and mortality in the two populations.
    e. "At-risk" children: Children with an index ED visit (the first ED visit for each child in the longitudinal ED cohort from 2012-2017) resulting in admission, transfer to another hospital for admission, or death. These children were used as the denominator (as opposed to all ED visits) to apply the observed and adjusted mortality found previously.[1] At-risk children have an elevated risk of death and are sensitive to different levels of ED pediatric readiness. Overall, these ED visits represent 4% of all pediatric visits to EDs, with considerable variable between EDs.
    f. Deaths under current levels of ED pediatric readiness: the estimated number of children 0-17 years presenting to EDs in a given state and dying during the same acute care visit (ED or inpatient) each year based on 2021 levels of ED pediatric readiness (2021 was the most recent national assessment), the distribution of at-risk ED visit volumes across the four quartiles of ED readiness, and our estimates for mortality.
    g. Deaths if all EDs were at high readiness: the estimated number of children 0-17 years presenting to EDs in each state and dying during the same acute care visit each year under a scenario of universally high ED pediatric readiness. To generate these estimates, we used the same methodology described in Section 1.f., and the risk-adjusted reduction in mortality for high-readiness EDs compared to current levels. This is the preventable mortality attributable to low ED readiness (quartiles 1-3). The difference between 1.f. and 1.g. represents the number of "lives that could be saved" annually in each state based on gaps in ED pediatric readiness.
    h. State vs. national estimates: We calculated state-by-state estimates and summed them to generate national estimates for the annual number of pediatric deaths among children presenting for emergency care and the number of pediatric deaths under universally high ED readiness (i.e., the difference representing the number of pediatric lives that could be saved nationally).
    i. Cost differential for all EDs to reach high readiness: Based on the estimated distribution of ED pediatric readiness and ED pediatric volume across all U.S. EDs, we calculated the total cost to achieve high readiness from current levels by state and across the US based on previously published data[2].

2. **Objectives**

We used a variety of data sources to estimate the state and national costs of raising all EDs to high pediatric readiness from current levels and the resulting annual number of pediatric lives that could be saved through this investment. We first identified all EDs currently functioning in the U.S., their levels of ED pediatric readiness, annual ED pediatric volume, and annual number of at-risk children. Using our cost estimates (Remick et al. JACEP. 2024), we calculated the state-specific annual costs for all EDs to reach and sustain high ED pediatric readiness. Based on our mortality data and risk-adjusted estimates, we also calculated the state-specific annual number of pediatric lives saved from universally high ED pediatric readiness. We summed the state-by-state cost and mortality estimates to yield national estimates, including measures of uncertainty.

© 2024 Newgard CD et al. *JAMA Network Open*.

3. **Data Sources**
   a. Distribution of EDs by state, levels of pediatric readiness (wPRS quartile), and annual ED volume of children (<1,800; 1,800-4,999; 5,000-9,999; ≥10,000).
      i. We created a list of all currently operating EDs in the U.S., their wPRS, and annual ED volume using the 2021 National Pediatric Readiness Project (NPRP) national assessment of ED pediatric readiness.[3] When missing, we used data from the 2013 NPRP national assessment.[4] The wPRS was calculated similarly and the ED volume categories were identical in both national assessments.
      ii. For EDs not completing the 2013 or 2021 NPRP surveys, we developed a hospital-level multiple imputation model to impute wPRS and pediatric ED volume using matched American Hospital Association (AHA) data.
         1. Creation of a sample of U.S. EDs that did and did not respond to either NPRP national assessment using a starting sample n = 5,150 hospitals invited to participate in the 2021 NPRP assessment.
            a. Using an existing crosswalk between AHA and NPRP respondents, we matched ED and hospital characteristics from the AHA data based on zip code, street address, and hospital name. EDs that did not match on all three items were subject to manual review to determine match success (e.g., use of PO Boxes, name change, etc.).
            b. We examined the AHA variable of service type and excluded EDs that were not eligible to be included (e.g., psychiatric, rehabilitation, specialty hospitals, and long-term acute care hospitals) and created a list of verified EDs with and without NPRP data. We also removed duplicate ED listing, EDs outside the US (i.e., in U.S. territories),
         2. Development of hospital-level multiple imputation model to estimate missing wPRS and annual ED pediatric volume for EDs that did not respond to the 2021 or 2013 national assessments (n = 289 EDs).
            a. We used PROC MI (SAS) to multiply impute a transformed continuous wPRS and categorical pediatric ED volume with the appropriate number of imputations and transformations of continuous variables for normality. AHA hospital predictors included:
               i. Total ED annual volume (continuous)
               ii. Pediatric ED volume (categorical)
               iii. CBSA Type (Metro, Micro, Rural) [Combined with NPRP Urbanicity when CBSA type missing]
               iv. Pediatric ED (yes/no)
               v. Hospital ownership (For profit, government, not for profit)
               vi. Trauma center (level 1-2, level 3-5, non-trauma centers)
               vii. Council of Teaching Hospitals
               viii. AMA medical school
               ix. ACGME
               x. State
               xi. Any Pediatric inpatient beds
               xii. Any pediatric ICU services
               xiii. Any neonatal beds
               xiv. Critical Access Hospital
               xv. Children's hospital
            b. We also developed a sensitivity MI model to assess use of 2013 NPRP assessment data when 2021 NPRP data was missing
      iii. We created a distribution of all U.S. EDs (50 states and D.C.) based on the four quartiles of wPRS and four categories of ED pediatric volume = 16 categories.

© 2024 Newgard CD et al. *JAMA Network Open*.

b. Distribution of at-risk children presenting to all U.S. EDs based on the four quartiles of wPRS and four categories of ED pediatric volume (i.e., annual number of at-risk children across the 16 categories).
   i. We used the total number of at-risk children presenting to all EDs in 11 states over 6 years (2012-2017), stratified by wPRS quartile and ED pediatric volume categories based on the initial ED visit[1]
   ii. Calculated average annual estimates for number of at-risk children using most recent years (2016-2017).
   iii. These data included the number of pediatric deaths among at-risk children presenting to these EDs (deaths in ED and inpatient following admission from ED) for each of the 11 states.

c. Data sources to validate estimates
   i. Annual number of at-risk children 0-17 years by state and year (admissions through the ED, as available in HCUP for 14 states); 8 of these states were unique from the original 11-state cohort.
      1. https://datatools.ahrq.gov/hcupnet/ (most recent data = 2020)
      2. Used 2019 data as the most recent and representative year because of major changes to ED use in 2020 due to the COVID-19 pandemic
   ii. Annual number of deaths in the ED and inpatient following admission from the ED for children 0-17 years by state and year, representing the total number of pediatric deaths among children presenting to EDs each year in 50 states.
      1. https://wonder.cdc.gov/controller/datarequest/D158 (this is the CDC WONDER database for all deaths by state and nationally)
      2. Used 2019 data as the most recent and representative year
      3. Because WONDER does not delineate source of admission for inpatient deaths, we used the ratio of children admitted through the ED who died inpatient vs. all pediatric inpatient deaths, as available from our 11-state cohort (average = 10.8%). We applied this ratio to the total number of pediatric inpatient deaths and added to the number of pediatric deaths in the ED to generate the total number of annual deaths among children presenting for emergency care.

4. Calculation of Estimates

a. Population of interest was state-level annual number of at-risk children in each of 50 states and DC. This population was used to estimate the annual number of pediatric deaths among children receiving emergency services.
   i. Strategy: we created a distribution of annual number of at-risk children across 16 categories for wPRS quartile x ED pediatric volume categories using our data from 11 states,[1] then applied this distribution to the known number of EDs in each state and their known 2021 level of ED pediatric readiness and ED volume. We tested four different techniques to generate metrics for the 11 states that would be generalizable to the other 39 states. We selected the ideal method based on validation using the above data sources. See Section 6 for the three methods trialed, but not used.
   ii. Method used:
      1. Given all at-risk ED visits by children in the 11-state cohort during 2016-2017 (most recent two years from these data[1]), we calculated the median annual at-risk ED visits by wPRS quartile and pediatric ED volume categories (= 16 categories), then applied this distribution to EDs in each state (including the 11 states used to generate the distribution).
      2. Using the observed distribution created above (eTable 1), we applied the distribution to EDs across all 50 states based on their known wPRS quartile and ED volume categories. The resulting values represented the estimated number of at-risk children seen in EDs in each state by year.
   iii. Methods for validation

1. We compared the estimates generated above to the validation data sources described in Section 3.c.

b. Estimated number of deaths among at-risk children given current level (2021) of ED pediatric readiness. Given the observed 11-state data, we calculated the model-based probability of mortality by wPRS quartile and ED pediatric volume category using predictive margins in Stata. We used these probabilities to generate the estimated number of deaths for each state based on the total number of EDs, their 2021 level of ED pediatric readiness, and annual ED pediatric volume (termed Measure 1). Corresponding SEs were calculated for all measures of mortality.
   i. Methods for validation
      1. CDC WONDER estimates, as detailed in Section 3.c.

c. Estimated number of deaths among at-risk children under a scenario of universally high ED readiness (all EDs at wPRS ≥ 88).
   i. Using risk-adjusted model estimates from our prior analyses[1] (11 states, separated by injury vs medical), we calculated the adjusted predicted mortality (predictive margins) for each quartile of readiness and the predicted mortality under the counterfactual case of all EDs being at the 4th quartile readiness (predictive margins in Stata). Corresponding SEs will be calculated for all measures of mortality.
   ii. For wPRS quartiles 1-3, we used the model-based predicted probability of mortality and the predicted probability at the highest quartile of readiness to estimate the percent reduction in mortality for EDs at each of wPRS quartiles 1, 2, and 3 if they moved to quartile 4 (high) ED readiness. We applied this percent reduction in mortality to each ED in each state based on their current level of ED readiness to estimate the number of pediatric deaths under the counterfactual scenario of universal high ED readiness (termed Measure 2).

d. Lives that "could be saved" if all EDs were high ready
   i. For each state, the difference between Measure 1 (current annual number of deaths among children presenting to EDs under current levels of ED readiness) and Measure 2 (annual number of deaths among children presenting to EDs under universally high ED readiness) represented the number of lives that could be saved if all EDs had high-readiness.
   ii. These estimates accounted for the level of wPRS, annual ED volume of children, number of EDs in each state, annual number of at-risk children in each state, and the risk-adjusted reduction in mortality expected from changing EDs from current levels to high readiness.

e. Cost to reach high readiness.
   i. Using our recently reported annual hospital-level costs (and corresponding uncertainty estimates) to reach and sustain high ED readiness based on current wPRS quartile and ED pediatric volume,[2] we estimated the state and national costs to reach high readiness. These estimates were based on the number of EDs in each state, 2021 level of ED readiness (wPRS), and known annual ED volume of children.
   ii. To calculate the total state-level annual cost to increase to high readiness, we multiplied the number of EDs in each wPRS/pediatric ED volume group by the hospital-level cost to reach high readiness. We calculated the total sum across the U.S. by summing across state estimates.
   iii. Converted \$2022 to \$2023 and calculated uncertainty estimates.

5. Calculating Uncertainty

a. Mortality
   i. Using the standard errors from the state-specific estimated number of deaths described above (Sections 4.b. and 4.c.), we calculated the standard error of deaths for each measure. We calculated these measures stratified by injury versus medical conditions, wPRS quartile, and ED pediatric volume across all states. Within each state, we calculated the total number of deaths as the sum across all strata. The estimate of uncertainty was calculated based on the distribution of the sum of these strata. For each state, we generated a bootstrapped sample of the estimated number of deaths given the calculated number of expected deaths and corresponding standard error. The standard

© 2024 Newgard CD et al. *JAMA Network Open*.

deviation of this distribution represented the standard error for that state's estimate. Confidence bands were defined as 1.96 * the standard error. The standard error for national-level estimates were generated using the same method, but across states instead of strata.

6. **Methods tested but not used for generating state-specific annual number of at-risk children using census data.**
    a. Created an overall ratio of at-risk ED visits by children across 11 states to the pediatric population in each of those states using the American Community Survey 5-year averages (census data) and extrapolated to the remaining 39 states using their 2021 pediatric population (issue: greatly overestimated annual number of at-risk children in smaller states and underestimated larger states).
    b. Created state-level ratios of at-risk ED visits by children across 11 states to the pediatric population (as above) and took the average of those ratios to extrapolate to the remaining 39 states using the 2021 pediatric population (issue: overestimated in smaller states and underestimated in larger states).
    c. Created state-level ratios of at-risk ED visits by children across 11 states within each quartile of wPRS to the pediatric population and took the average of those ratios to extrapolate to the remaining 39 states using the 2021 pediatric population (issue: overestimated in smaller states and underestimated in larger states)

eAppendix 2. Sensitivity analyses.

To test the heterogeneity of the association between high ED pediatric readiness and survival across states, we used an interaction term of state x ED pediatric readiness quartile. There was variability in the slopes of the interaction terms across states (using California as the referent state), but overall p-values indicated no significant interaction (all p values > 0.05).

To test the potential impact of using 2013 wPRS values in cases where 2021 values were missing, we used 2,490 EDs with both 2013 and 2021 values to impute wPRS using the 2013 value, then repeated all analyses. Compared to results from the primary analysis, 66% of EDs remained in the same quartile of ED pediatric readiness. For the 34% of EDs that changed wPRS quartile, 53% changed to a higher quartile and 47% to a lower quartile. Thus, there was an even distribution of values being imputed lower and higher than in the primary analysis. Using these values, there was <1% increase in the total number of estimated high acuity pediatric ED visits each year and an increase of 40 lives saved across the U.S. (~2% increase). Under the sensitivity analysis, the national costs to reach high ED readiness were $281,346 lower (<1% change). These comparisons suggest that using wPRS values from the 2013 assessment when 2021 values were missing was reasonable and produced conservative estimates for the number of lives saved and costs to achieve ED high readiness.

© 2024 Newgard CD et al. *JAMA Network Open*.

eReferences

1. Newgard CD, Lin A, Malveau S, et al. Emergency Department Pediatric Readiness and Short-term and Long-term Mortality Among Children Receiving Emergency Care. *JAMA Netw Open.* 2023;6(1):e2250941.

2. Remick KE, Gausche-Hill M, Lin A, et al. The hospital costs of high emergency department pediatric readiness. JACEP Open, 2024 June 03: 5(3), e13179. doi.org/10.1002/emp2.13179

3. Remick KE, Hewes HA, Ely M, et al. National Assessment of Pediatric Readiness of US Emergency Departments During the COVID-19 Pandemic. JAMA Netw Open 2023;6(7):e2321707. DOI: 10.1001/jamanetworkopen.2023.21707.

4. Gausche-Hill M, Ely M, Schmuhl P, et al. A national assessment of pediatric readiness of emergency departments. JAMA Pediatr 2015;169(6):527-34. DOI: 10.1001/jamapediatrics.2015.138.

© 2024 Newgard CD et al. *JAMA Network Open*.
