# Supplementary material for: State and National Estimates of the Cost of Emergency Department Pediatric Readiness and Lives Saved
Source: JAMA Netw Open. 2024 Nov 1;7(11):e2442154. doi: 10.1001/jamanetworkopen.2024.42154 (PMC11530936; doi:10.1001/jamanetworkopen.2024.42154)
Supplement: Supplement 2. — Data Sharing Statement [file jamanetwopen-e2442154-s002.pdf]

Data Sharing Statement

Newgard. State and National Estimates of the Cost of Emergency Department Pediatric Readiness and Lives Saved. *JAMA Netw Open*. Published November 01, 2024. doi:10.1001/jamanetworkopen.2024.42154

Data
Data available: No

Additional Information
Explanation for why data not available: The data are restricted by existing data use agreements, but much of these data are publicly available to researchers.
